# Softening with Ceramic Micro-Filtration for Application on Water Reclamation for Industrial Recirculating Cooling Systems

**DOI:** 10.3390/membranes12100980

**Published:** 2022-10-10

**Authors:** Noor Jehan Gulamussen, Daniël Donse, André Marques Arsénio, Sebastiaan Gerard Jozef Heijman, Louis Cornelis Rietveld

**Affiliations:** 1 Department of Water Management, Faculty of Civil Engineering and Geosciences, Delft University of Technology, 2628 CN Delft, The Netherlands; 2 Department of Chemistry, Faculty of Science, Eduardo Mondlane University, Maputo P.O. Box 257, Mozambique

**Keywords:** water softening, ceramic membranes, water reclamation, cooling systems

## Abstract

There is a global need for optimizing the use of water that has resulted from increased demand due to industrial development, population growth, climate change and the pollution of natural water resources. One of the solutions is to use reclaimed water in industrial applications that do not require water of potable quality, such as cooling water. However, for cooling water, (treated) wastewater’s hardness is too high, apart from having a high load of suspended solids and organic matter. Therefore, a combination of softening with ceramic micro-filtration was proposed for treating wastewater treatment effluent containing fouling agents for potential use in industrial cooling systems. The effectiveness of the softening process on model-treated wastewater with calcium hydroxide in the presence of phosphate and sodium alginate was first evaluated using jar tests. Furthermore, membrane fouling was studied when filtering the softened water. The results showed that the inhibition of calcium carbonate precipitation occurred when inorganic substances, such as phosphate and organic compounds, were present in the water. The fouling of the membranes due to sodium alginate in water was only slightly negatively affected when combined with softening and phosphate. Therefore, this combination of treatments could be potentially helpful for the post-treatment of secondary effluent for cooling systems.

## 1. Introduction

Water shortages in arid and semi-arid areas are drivers for the use of reclaimed water in industry [1]. Even though the costs of advanced treatment processes for contaminant removal are high, they can be justified by the high value of reclaimed water compared with the use of the scarce natural water sources [2].

Several power plants in the US have already used secondary-treated municipal wastewater treatment plant (WWTP) effluent as make-up water in their recirculating cooling water [3,4]. However, since WWTP effluent typically contains considerable concentrations of hardness, phosphate, ammonia, dissolved solids, and organic matter compared with the concentrations in fresh surface water, for example, extensive treatment is needed [5].

A cooling system relies on water as a heat transfer medium and is the most water-demanding process in industry [6]. Water is utilized to cool pumps and compressors of vacuum systems and steam turbine condensers. Cooling systems can have different configurations but are mainly divided into once-through and recirculating systems [7]. Once-through cooling systems transfer process heat to water to cool the process equipment and then discharge the hot water after a single use. This system requires a large volume of water; typically lake, river-, and seawater are used with little or no treatment [8]. Recirculating cooling systems transfer the heat from warmed water to vapor so that the water can be reused to absorb process heat and recirculated for additional cycles [9,10]. However, as evaporation occurs, the concentration of mineral salts increases, and when the concentration of mineral salts exceeds their solubility, scale formation on heat exchanger surfaces may occur. The level of dissolved solids (mineral salts) is controlled by discharging part of the recirculating water, called blowdown water, from the system and replenishing this volume with fresh make-up water. Previous studies have shown that the major mineral scales formed in recirculating cooling systems using WWTP effluent as make-up water are calcium carbonate and, to a lesser extent, calcium phosphate [5]. To avoid scale formation, hardness in the make-up water should be lower than 1.25 mmol L^−1^ [11]. Therefore, when using WWTP effluent as cooling water make-up water, additional treatment, such as filtration, chemical precipitation, ion exchange, or reverse osmosis, may be necessary [12].

Ceramic micro-filtration (CMF) is a potential treatment alternative for water reclamation [13]. Ceramic membranes, compared with the most commonly used polymeric membranes, are robust, have high mechanical strength [14], high chemical and thermal resistance [15], high membrane porosity, membrane permeability, and a homogeneous distribution of narrow pores [16]. Ceramic membranes can also be used at a higher flux than polymeric micro-filtration membranes and, therefore, reduce the membrane surface area needed for the same quantity of wastewater [17]. Ceramic membranes are also expected to withstand damage by high pressure, high temperatures or chemicals, enabling processes such as vigorous chemical cleaning of the membrane [18]. Other benefits are the membrane’s long life and the membrane material’s recyclability [14,19]. The disadvantage of ceramic membranes is their relatively high costs compared with polymeric membranes but they could be compensated by the advantages mentioned earlier [20].

MFs are low pressure-driven separation processes that are less energy intensive than traditional treatment methods [19]. They can be used to remove microorganisms and suspended or colloidal particles. However, they do not remove dissolved substances [21]. Nevertheless, previous studies have shown that softening can be promoted by the precipitation of calcium ions (Ca^2+^) in a membrane system [22,23]. Calcium carbonate particles are formed by dosing a base, and these inorganic particles can be removed from the water by membrane filtration. The findings showed that in a system containing Ca^2+^ and bicarbonate better softening was achieved in the absence of phosphate (PO_4_^3−^) due to the inhibitive effect of PO_4_^3−^ on calcium carbonate crystal growth. On the other hand, it is known that calcium promotes the complexation of organic matter, thereby influencing the rate of flux decline in membranes [24].

Therefore, in this work, attention was given to developing a novel application of CMF treatment, focusing on the removal of hardness in model WWTP effluent containing fouling agents, such as Ca^2+^, Mg^2+^, HCO_3_^−^, and organic matter typically present in WWTP effluent. The effectiveness of calcium hydroxide (Ca(OH)_2_) as a softening agent was studied during softening with CMF membranes in the presence of PO_4_^3−^. In addition, the effect of organic compounds on the softening process was analyzed using model water with sodium alginate (SA).

## 2. Materials and Methods

### 2.1. Experimental Setup

To study the precipitation of calcium carbonate followed by CMF, in the presence of potentially interfering inorganic and organic substances, two configurations were used:

1. Jar tests to rapidly evaluate the effectiveness of the softening agent in the presence of PO_4_^3−^ and SA. Solutions containing Ca^2+^, HCO_3_^−^, PO_4_^3−^, and SA were prepared in demineralized water.
2. Membrane tests to study the performance of the membrane when filtering precipitated calcium carbonate in combination with PO_4_^3−^ and SA, all prepared with demineralized water.

Ca(OH)_2_ in suspension, a common base employed in the softening process [25], was used in this work.

The concentrations of Ca^2+^ (1.5–3 mmol L^−1^) and PO_4_^3−^ (0.1 mmol L^−1^) used during the experiments represent the values found in WWTP effluent [26,27,28]. The concentration of SA that was previously used to simulate the concentration of organic substances in non-treated domestic wastewater is approximately 0.8 g L^−1^ [27,29,30]. However, to avoid too rapid clogging, only half of the concentration (0.4 g L^−1^) was used during the experiments.

A chemical cleaning with citric acid (1.5%) and chlorine (0.1%) was performed after the first set of membrane experiments and then after each membrane experiment (according to protocols presented in the sections below), and all experiments were executed in duplicate.

### 2.2. Jar Tests

Precipitation tests were performed in jars to study the differences between the conditions, with and without PO_4_^3−^ and SA. Table 1 presents the tested conditions. For each condition, samples were collected and filtered over a 0.45 µm pore filter (Whatman, Germany) in order to determine the remaining Ca^2+^ concentration using ion chromatography (IC, ProfIC 15–AnCat ion chromatograph Metrohm 881 anion). An A Supp 150/4.0 anion column was used with 3.2 mmol L^−1^ Na_2_CO_3_ and 1 mmol L^−1^ NaHCO_3_ eluent. To calibrate the IC, 6 standard solutions of Ca^2+^ (0.0025, 0.025, 0.25, 1.25, 2.5, and 3.75 mmol L^−1^) were used.

Due to the inhibitive nature of PO_4_^3−^, its effect was further analyzed considering different dosings of Ca(OH)_2_ (1.5 and 2.5 mmol L^−1^).

### 2.3. Membrane Tests

#### 2.3.1. Effect of Softening Agent on Fouling

**Figure 1:**
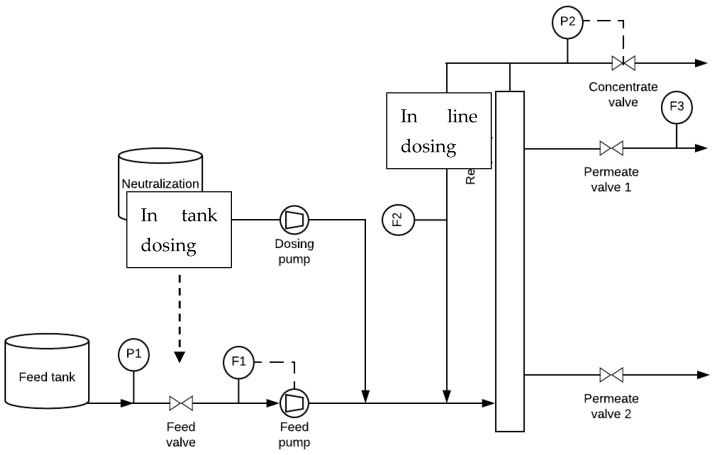
Schematic representation of the ceramic micro-filtration (CMF) setup. F1—feed flow meter (0–300 L h^−1^ ± 1 L h^−1^). F2—recycle flow meter (0–1000 L h^−1^). F3—permeate flow meter (0–30 L h^−1^). P1: pressure meter (0–20 ± 0.1 bar). P2—pressure meter (0–20 ± 0.1 bar).

The setup depicted in Figure 1 was used for in-line base and in-tank dosings from a neutralization tank to the membrane system.

The first two experiments consisted of:

B1 Filtration of the solution containing only a mixture of 3 mmol L^−1^ of Ca^2+^ and 6 mmol L^−1^ of HCO_3_^−^ to draw the base line and confirm that there is no retention of Ca^2+^ on the membrane.

B2 Filtration of the solution containing a mixture of 3 mmol L^−1^ of Ca^2+^ and 6 mmol L^−1^ of HCO_3_^−^ with a constant dosage of Ca(OH)_2_ (2.5 mmol L^−1^) to remove the hardness.

The feed flow was 25–30 L h^−1^ at a constant (transmembrane) pressure of 2 bar and the dosing pump constantly added 3 L h^−1^ of Ca(OH)_2_ with a concentration of 22.73 mmol L^−1^ Ca(OH)_2_, which resulted in a concentration of 2.5 mmol L^−1^ Ca(OH)_2_ in the feed flow. The recovery fluctuated approximately 75–80%. The specification of the used membranes is presented in Table 2.

For each experiment, samples of the feed water, permeate, and concentrate flow were taken in duplicate at three points during the tests, after 15 min, 1 h, and at the end of the experiment, respectively. The samples were analyzed for the concentration of Ca^2+^ using IC.

No flux decline was observed during the two experiments.

After the first set of experiments, the configuration was changed from in-line dosing to in-tank precipitation, where the base was added in the influent tank for the rest of the experiments.

#### 2.3.2. Individual Effect of Sodium Alginate, Calcium, and Softening on Membrane Fouling

The following set of experiments consisted of evaluating the fouling of CMF in the presence of SA, Ca^2+^, and HCO_3_^−^ with and without the addition of Ca(OH)_2_, using membranes in the same condition leading to a similar initial flux.

The tested conditions are presented in Table 3.

For the experiments, the cleaning followed the same procedure as described in the previous section, except for substituting the citric acid with a solution of sodium hypochlorite 0.1% to remove the organic fouling better [27].

#### 2.3.3. Combined Effect of Sodium Alginate, Phosphate, and Softening on Fouling

The last set of experiments consisted of evaluating the fouling of CMF in the presence of Ca^2+^, HCO_3_^−^, PO_4_^3−^ and SA, with the addition of Ca(OH)_2_, compared with a solution consisting of SA only, using membranes in the same condition leading to a similar initial flux.

The tested conditions are presented in Table 4.

For the experiments, the cleaning followed the same procedure as described in the previous section.

#### 2.3.4. Flux Recovery

During the membrane experiments in the presence of organic fouling (SA), the initial flux varied, indicating that some irreversible fouling occurred during filtration and/or the cleaning protocol did not function efficiently. The membrane flux recovery is the ratio of pure water flux after a filtration and cleaning process, *J_pwfn_*, to the pure water flux of the first filtration, *J_pwf_*_1_, after either hydraulic or chemical washing.

The flux recovery is calculated using Equation (1) below:

(1) $$R=\frac{J_{pwfn}}{J_{pwf1}}$$

## 3. Results

### 3.1. Jar Tests of Softening Process and Influence of PO_4_^3−^ and Organic Compounds

#### 3.1.1. Effect of Ca(OH)_2_ as Softening Agent

**Figure 2:**
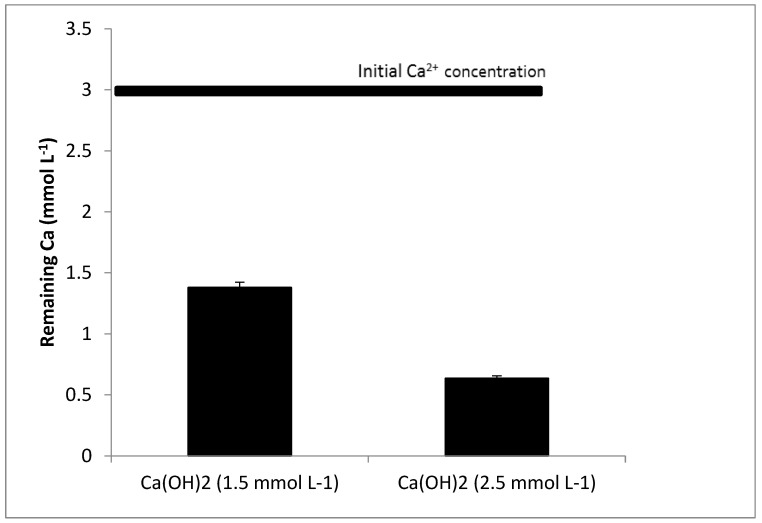
Calcium removal with calcium hydroxide.

Figure 2 shows the results of the remaining Ca^2+^ after the precipitation with Ca(OH)_2_. When the concentration of Ca(OH)_2_ was low (1.5 mmol L^−1^), the removal was relatively high as, with the addition of 1.5 mmol L^−1^, it was expected that the concentration of Ca^2+^ would be reduced to half (from 3 to 1.5 mmol L^−1^). The fact that Ca(OH)_2_ is partially soluble (Kps = 5.5 × 10^−6^) [31] and was added to the water as a suspension enhanced the nucleation and stimulated the crystallization process. The original solution was most likely already somewhat supersaturated with CaCO_3_ [32]. When the concentration of Ca(OH)_2_ was increased to 2.5 mmol L^−1^, the removal increased, but not linearly. Two explanations can be given: (1) At low supersaturation, the nucleation mechanism is heterogeneous, whereas, at higher supersaturation, homogeneous nucleation prevails [33]. The homogeneous nucleation results in an increasing number of formed nuclei. These nuclei have a relatively lower chance of growing to large crystals compared with the growth of a lower number of formed nuclei during heterogeneous crystallization [34]. Therefore, precipitation is hampered by an excessive increase in Ca(OH)_2_ concentration. (2) A CaCO_3_ film is developed on the Ca(OH)_2_ particles and will eventually be partially or entirely enclosed. This film can inhibit Ca(OH)_2_ from dissolving any further by a layer of CaCO_3_ formed on the surface of a particle in water, called the dissolve-precipitate mechanism [35].

#### 3.1.2. Effect of Phosphate and Sodium Alginate on Softening

**Figure 3:**
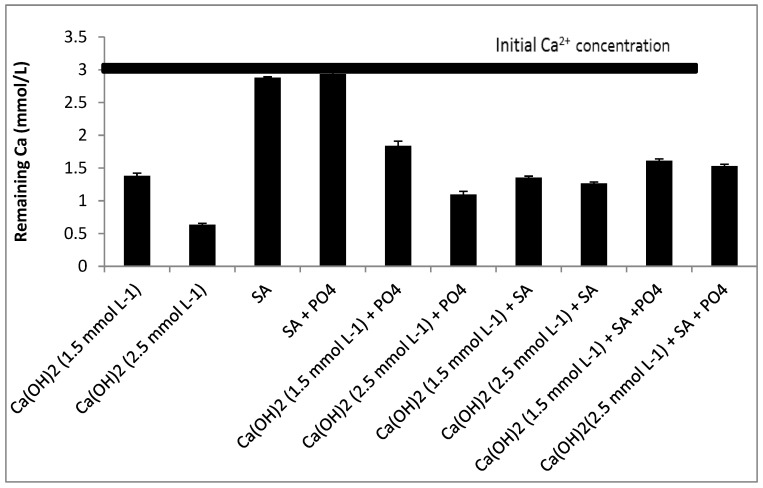
Effect of phosphate and sodium alginate in softening with calcium hydroxide.

Both PO_4_^3−^ and SA inhibited the precipitation of CaCO_3_ Figure 3. Inhibition by SA was observed only when the concentration of Ca(OH)_2_ was increased to 2.5 mmol L^−1^ Figure 3. With a lower dosage of Ca(OH)_2_ (1.5 mmol L^−1^) the inhibition was dominated by the presence of PO_4_^3−^ rather than by SA, when considered separately. This was also found earlier, when the growth of CaCO_3_ took place in the presence of PO_4_^3−^ and interfered with the CaCO_3_ crystal growth process because of interactions with the lattice ions of CaCO_3_ at the respective active crystal growth sites [36].

Two processes have been accepted to explain the mechanisms of potential inhibition by organic molecules. First is the formation of chelate complexes of dissolved Ca^2+^ ions with organic molecules, which reduces the effective supersaturation of CaCO_3_, thereby decreasing the rate of nucleation and crystal growth, depending on the saturation state [37]. Second is the adsorption of organic compounds on the specific surface of the CaCO_3_ crystals. The active growth sites on the CaCO_3_ surface may be blocked by the adsorption reactions, thus preventing the further growth of the crystals of CaCO_3_ [38,39]. The negative charge of SA, due to deprotonated carboxylic functional groups, may then induce repulsive inter- and intramolecular electrostatic forces, decreasing the chances of CaCO_3_ precipitation [24].

When phosphate and SA were considered simultaneously, the precipitation of Ca^2+^ was less influenced compared with the condition where PO_4_^3−^ was considered separately, especially when the concentration of Ca(OH)_2_ was 1.5 mmol L^−1^. The chelate complexes formed after adding SA may thus have masked Ca^2+^ and inactivated the interaction between Ca^2+^ and PO_4_^3−^ [37].

#### 3.1.3. Extent of Inhibition Effect of Phosphate

**Figure 4:**
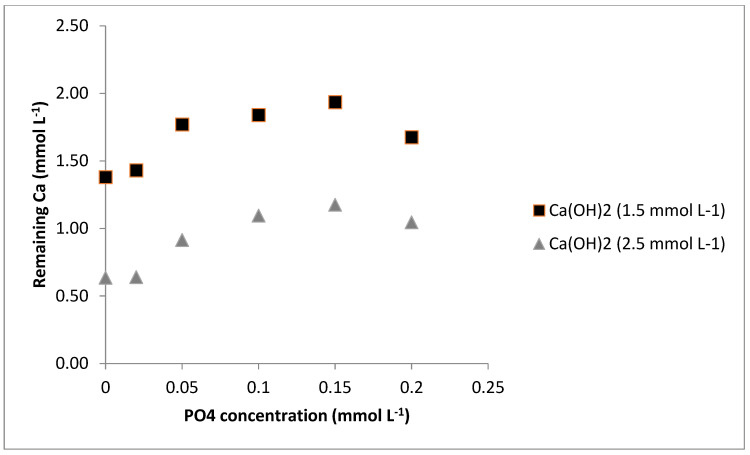
Inhibition effect of PO_4_^3−^ on precipitation of CaCO_3_.

From Figure 4, it can be observed that an increment in the concentration of PO_4_^3−^ from 0.05 to 0.15 mmol L^−1^ led to a small increase in the inhibition of CaCO_3_ precipitation, but it was slightly reduced when 0.2 mmol L^−1^ of PO_4_^3−^ was added. Further, when the concentration of Ca(OH)_2_ was increased from 1.5 to 2.5 mmol L^−1^, the effect of PO_4_^3−^ was similar. Gebauer et al. [40] studied the role of additives in CaCO_3_ precipitation. They also found that the addition of 10 mg/L sodium triphosphate (approximately 0.1 mmol L^−1^) solution performed only slightly weaker than the addition of 100 mg/L sodium triphosphate (1 mmol L^−1^). Therefore, this suggests that the mechanism of colloidal stability of the intermediate cluster structures is the most relevant in suppressing nucleation in this system and not the stoichiometric binding events (as expected by an ion-binding-like mechanism).

### 3.2. Ceramic Membrane Tests Results

#### 3.2.1. Effect of Softening on Fouling

During the CMF tests without adding a base, the IC results (Table 5) showed a constant Ca^2+^ feed concentration of 1.6 mmol L^−1^. The first two samples of the retentate were not taken because too little was produced. The retentate showed values of approximately 0.1 mmol L^−1^ lower than the feed. Since the base was not added in this experiment, no difference in concentration was expected from the collected samples, as the pores of the CMF are too large to retain dissolved salts (i.e., Ca(HO_3_)_2_) without previous precipitation [41], which was confirmed by the obtained results.

In the experiment with the addition of Ca(OH)_2_, the Ca^2+^ concentration in the feed flow was approximately 1.9 mmol L^−1^ (Table 5). The chemical dosing in this experiment was 2.5 mmol L^−1^ of Ca(OH)_2_, directly dosed into the feed flow. The Ca^2+^ concentration in the retentate decreased to a value of 0.5 mmol L^−1^ and, near the end of the experiment, even reached a concentration of less than 0.1 mmol L^−1^, having precipitated 1.4 to 1.8 mmol L^−1^. Since the base was stoichiometrically overdosed, it was expected that almost all Ca^2+^ would be removed by precipitation. These results were in accordance with the results of the jar tests, where we found that when increasing the concentration of Ca(OH)_2_ to 2.5 mmol L^−1^, substantial removal of Ca^2+^ was observed. The results were also in accordance with the results of Zeppenfeld [42] and Heinsbroek [22], who found that by increasing the carbonate concentration, promoted by the addition of the base, thereby increasing supersaturation, the rate constant increased linearly and consequently increased the Ca^2+^ removal.

**Figure 5:**
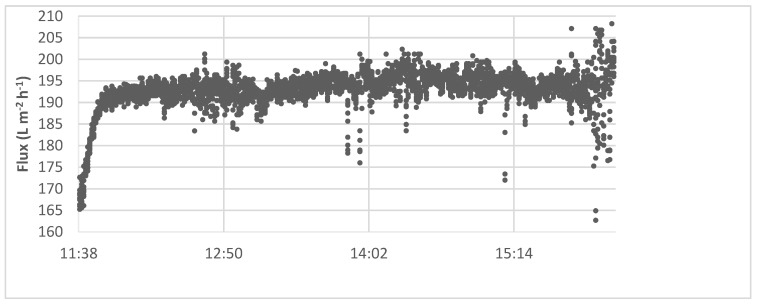
Membrane performance of the softening with calcium hydroxide.

During the experiment, the flux built up rapidly in the first few minutes of the test. Still, it stayed near a constant value (of 190 L m^−2^ h^−1^) for the rest of the experiment, which is represented by the example in Figure 5, indicating that scaling of the membrane did not occur during the experiment [22].

#### 3.2.2. Individual Effect of Sodium Alginate, Calcium, and Softening Agent on Fouling

**Figure 6:**
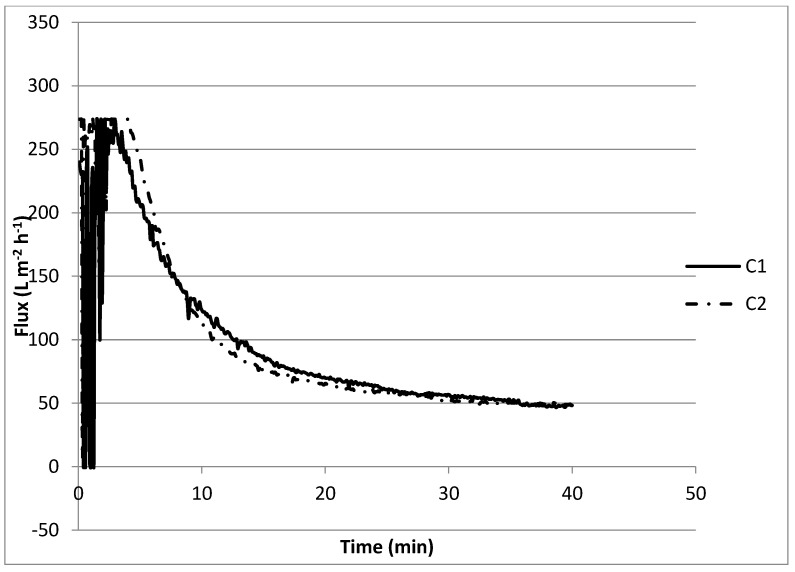
Membrane performance for the effect of sodium alginate, calcium, and softening agent on fouling.

When filtering a solution with Ca^2+^, bicarbonate, and 0.4 g L^−1^ of SA, the initial retentate flux was 270 L m^−2^ h^−1^, pronouncedly decreased, and reached a flux of approximately 50 L m^−2^ h^−1^ in more than 30 min (experiment C1) (Figure 6). The accentuated decrease in the retentate flux can be explained by the complexation of Ca^2+^ with alginate, forming a compacted gel layer on the membrane surface. Compression of the electric double layers of the alginate on the membrane results in a lower electrostatic repulsion and a denser fouling layer with a higher absolute resistance [43,44]. Not much difference (the flux also reached 50 L m^−2^ h^−1^ in more than 30 min) was observed when 1.5 mmol L^−1^ Ca(OH)_2_ was added to the previous mixture (experiment C2). From the jar tests, it was observed that, approximately 50% of Ca^2+^ was removed in the presence of SA, and apparently, this removal did not influence the performance of the membrane, e.g., through scaling.

#### 3.2.3. Combined Effect of Sodium Alginate, Calcium, Phosphate, and Softening Agent on Fouling

**Figure 7:**
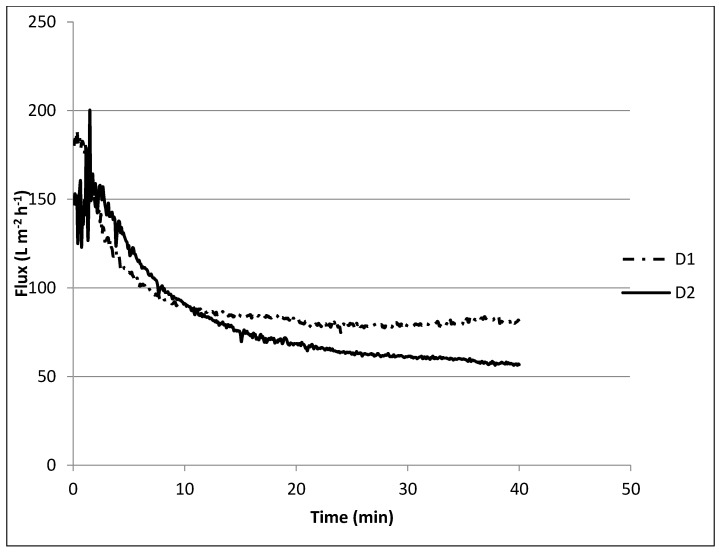
Membrane performance for the combined effect of sodium alginate, calcium, phosphate, and softening agent on fouling.

During experiment D1, where a solution containing only 0.4 g L^−1^ SA was filtered, it took approximately 10 min to lower the retentate flux from 150 L m^−2^ h^−1^ to approximately 80 L m^−2^ h^−1^, 2/3 (Figure 7) and then stayed more or less constant. In all experiments, a steep decline in permeability at the beginning of filtration was observed and was most likely caused by the loading effect [45] being a direct interaction between the membrane surface and the alginate molecules that could then create a firm fouling layer. This interaction is mostly dominated by the adsorption of foulant to the membrane surface, which leads to pore constriction [24].

Similar behavior was observed when dosing Ca(OH)_2_ in the presence of SA, Ca^2+^, HCO_3_^−^, and PO_4_^3−^ (D2), although the final flux further decreased to approximately 60 L m^−2^ h^−1^. This could be explained by the complexation of Ca^2+^ with alginate forming a compacted gel layer on the membrane surface and some scaling of CaCO_3_ on the surface or in the pores [46]. Although flux decline was low compared with the first experiment, it is likely that the Ca^2+^ removal was negatively affected, as was observed during the jar tests.

#### 3.2.4. Flux Recovery Results

From Figure 6 and Figure 7 it can be observed that the initial flux declined from 270 to 200 L m^−2^ h^−1^, a flux recovery of 74%. These results are in accordance with the first set of experiments (Figure 5) with recoveries between 75 and 80%. Katsoufidou et al. [47] also analyzed a fouling of membranes with SA and found that the fouling was due to two mechanisms; (1) a rapid irreversible fouling due to internal pore constriction, followed by (2) cake development on the membrane surface, which becomes the dominant fouling mechanism when calcium concentration increases. This may explain why we observed a low flux recovery in the last experiments.

## 4. Conclusions

A novel method of treatment of the model WWTP effluent with ceramic micro-filtration (CMF) for use in cooling systems was developed with a focus on scale formation by removing hardness in the presence of other fouling agents, such as organic matter. The effectiveness of Ca(OH)_2_ as a softening agent was studied using jar tests, and the effect of organic compounds on the softening process was analyzed using model water with SA. During the CMF tests, the effect on fouling (through flux decline) was studied.

It was found that PO_4_^3−^ inhibited the precipitation of CaCO_3_ when using Ca(OH)_2_ as a softening agent. In conditions where PO_4_^3−^ was present, the concentration of Ca(OH)_2_ needed to be increased to compensate for the precipitation of PO_4_^3−^ and the inhibition of the calcite crystallization process. Organic compounds also affected the removal of Ca^2+^ on the softening process, although in the presence of PO_4_^3−^, the alginate inhibition of the crystallization process was reduced.

Finally, it was concluded that the fouling of the membranes due to sodium alginate in water was only slightly negatively affected by PO_4_^3−^ and when combined with softening.

## Figures and Tables

**Table 1:** Jar tests softening conditions.

Experiments	Ca^2+^ (mmol L^−1^)	HCO_3_^−^ (mmol L^−1^)	Ca(OH)_2_ (mmol L^−1^)	PO_4_^3−^ (mmol L^−1^)	Sodium Alginate (g L^−1^)
A1	3	6	1.5	0	0
A2	3	6	2.5	0	0
A3	3	6	0	0	0.4
A4	3	6	0	0.1	0.4
A5	3	6	1.5	0.1	0
A6	3	6	2.5	0.1	0
A7	3	6	1.5	0	0.4
A8	3	6	2.5	0	0.4
A9	3	6	1.5	0.1	0.4
A10	3	6	2.5	0.1	0.4

**Table 2:** Ceramic micro-filtration (CMF) membrane specification (Inopor GmbH).

Material	Al_2_O_3_
Surface area	0.11 m^2^
Diameter of membrane	25.4 mm
Diameter of tubes	7 mm
Number of tubes	4
Length	1200 mm
Direction of flow	Crossflow
Nominal permeability at 1 bar	25 L m^−2^ h^−1^
Pore size	0.1 µm

**Table 3:** Effect of sodium alginate, calcium, and softening on fouling.

Experiments	Ca^2+^ (mmol L^−1^)	HCO_3_^−^ (mmol L^−1^)	Ca(OH)_2_ (mmol L^−1^)	PO_4_^3−^ (mmol L^−1^)	Sodium Alginate (g L^−1^)
C1	3	6	0	0	0.4
C2	3	6	1.5	0	0.4

**Table 4:** Effect of organic compounds on softening.

Experiments	Ca^2+^ (mmol L^−1^)	HCO_3_^−^ (mmol L^−1^)	Ca(OH)_2_ (mmol L^−1^)	PO_4_^3−^ (mmol L^−1^)	Sodium Alginate (g L^−1^)
D1	0	0	0	0	0.4
D2	3	6	1.5	0.1	0.4

**Table 5:** Calcium concentrations in the feed, permeate, and concentrate samples.

	Ca^2+^ Feed (mmol L^−1^)	Ca^2+^ Permeate (mmol L^−1^)			Ca^2+^ Retentate (mmol L^−1^)		
Time (hours)		0.25	1	6	0.25	1	6
Baseline	1.62	1.54	1.65	1.61	-	1.49	1.47
Added Ca(OH)_2_ (2.5 mmol L^−1^)	1.92	0.48	0.51	0.07	0.44	0.53	0.13
